# Impact of inadequate adherence on response to subcutaneously administered anti-tumour necrosis factor drugs: results from the Biologics in Rheumatoid Arthritis Genetics and Genomics Study Syndicate cohort

**DOI:** 10.1093/rheumatology/keu358

**Published:** 2014-09-10

**Authors:** James Bluett, Catharine Morgan, Layla Thurston, Darren Plant, Kimme L. Hyrich, Ann W. Morgan, Anthony G. Wilson, John D. Isaacs, Lis Cordingley, Anne Barton

**Affiliations:** ^1^Arthritis Research UK Centre for Genetics and Genomics, University of Manchester, ^2^National Institute of Health Research Manchester Musculoskeletal Biomedical Research Unit, Central Manchester Foundation Trust and University of Manchester, Manchester Academic Health Science, ^3^Arthritis Research UK Centre for Epidemiology, University of Manchester, Manchester, ^4^Leeds Institute of Rheumatic and Musculoskeletal Medicine, University of Leeds, National Institute of Health Research Leeds Musculoskeletal Biomedical Research Unit, Leeds, ^5^Academic Unit of Rheumatology, Department of Infection and Immunity, University of Sheffield Medical School, Sheffield, ^6^Musculoskeletal Research Group, Institute of Cellular Medicine, Newcastle University and National Institute of Health Research Newcastle Biomedical Research Centre, Newcastle upon Tyne and ^7^Institute for Inflammation and Repair, University of Manchester, Manchester, UK.

**Keywords:** rheumatoid arthritis, biologic therapies, behaviour, adherence, outcome measures

## Abstract

**Objective.** Non-adherence to DMARDs is common, but little is known about adherence to biologic therapies and its relationship to treatment response. The purpose of this study was to investigate the association between self-reported non-adherence to s.c. anti-TNF therapy and response in individuals with RA.

**Methods.** Participants about to start s.c. anti-TNF therapy were recruited to a large UK multicentre prospective observational cohort study. Demographic information and disease characteristics were assessed at baseline. Self-reported non-adherence, defined as whether the previous due dose of biologic therapy was reported as not taken on the day agreed with the health care professional, was recorded at 3 and 6 months following the start of therapy. The 28-joint DAS (DAS28) was recorded at baseline and following 3 and 6 months of therapy. Multivariate linear regression was used to examine these relationships.

**Results.** Three hundred and ninety-two patients with a median disease duration of 7 years [interquartile range (IQR) 3–15] were recruited. Adherence data were available in 286 patients. Of these, 27% reported non-adherence to biologic therapy according to the defined criteria at least once within the first 6-month period. In multivariate linear regression analysis, older age, lower baseline DAS28 and ever non-adherence at either 3 or 6 months from baseline were significantly associated with a poorer DAS28 response at 6 months to anti-TNF therapy.

**Conclusion.** Patients with RA who reported not taking their biologic on the day agreed with their health care professional showed poorer clinical outcomes than their counterparts, emphasizing the need to investigate causes of non-adherence to biologics.

## Introduction

The development of biologic drugs that block the TNF pathway has revolutionized RA treatment and patient prognosis. Anti-TNF drugs reduce joint inflammation, diminish radiological damage and may reduce cardiovascular risk [1, 2]. There are currently four anti-TNF biologics administered subcutaneously that are licensed for treatment of RA within the UK. In the UK, eligibility for biologics is determined by guidance issued by the National Institute for Health and Care Excellence [3]. Eligibility to commence and maintain treatment with anti-TNF therapy is determined by the 28-joint DAS (DAS28) [4]. A score ≥5.1 on two separate occasions at least 1 month apart is required before UK patients are eligible for anti-TNF therapy, while a response ≥1.2 is required 6 months after initiation for biologic drugs to be continued.

The optimum efficacy of biologic drugs reported in randomized controlled trials may only occur if patients are fully adherent to their medication. Adherence is defined as ‘the extent to which the patient’s behaviour—taking medication, following a diet, and/or executing lifestyle changes, corresponds with agreed recommendations from a health-care provider’ [5]. Non-adherence can be considered either unintentional due to factors beyond the control of the patient, or intentional, in which patients choose not to take the drug or take it in a way that differs from the advice given [6]. Patients with RA are expected to adhere consistently and over a long period of time to a range of combinations of medications and have high levels of concern that associate with non-adherence [7, 8]. The consequences of low or non-adherence may include additional health care costs, an increased number of appointments, disease progression and more aggressive treatment with the potential for adverse events [9].

To date, there are four published studies of adherence to biologics in RA, with adherence rates based on a variety of different criteria ranging from 32% to 70% [10–13]. All investigated adherence to biologics within the USA utilizes prescription claims data, for which there are known disadvantages. Claims data are not collected as a research tool, but for payment processing, therefore a claim does not indicate that the patient took the medication. A period of no claim for a patient may not indicate non-adherence, but may be due to temporary cessation of medication due to an intercurrent illness such as infection. The majority of studies assessed adherence using the medication possession ratio (MPR), calculated as the percentage of days during the follow-up period that the patient had a supply of the medication. A cut-off MPR is used to determine adherence (typically 80%), however, the cut-off values are arbitrary rather than a clinically significant MPR that has been shown to affect response to treatment. Importantly, a major disadvantage of previous studies is that they have not tested the link between adherence rates and the subsequent impact on response to treatment. The European League Against Rheumatism (EULAR) has recommended that a future research agenda should include the question ‘how good is patient adherence to biological agents and can a lack of adherence be related to loss of efficacy [14].

The objectives of the present study were to describe current self-reported adherence to s.c. anti-TNF therapy in RA and to investigate the relationship between adherence and response to treatment in a cohort of patients with RA from the UK.

## Patients and methods

### Study design

Patients were recruited for this study from the prospective arm of the Biologics in Rheumatoid Arthritis Genetics and Genomics Study Syndicate (BRAGGSS) [15]. BRAGGSS is a multicentre study collecting clinical, laboratory and adherence data on patients with RA who are starting treatment with biologic therapy in the UK.

### Study participants

Patients attending 60 rheumatology clinics across the UK were recruited into the study between November 2008 and March 2012. Patients had RA as diagnosed by a consultant rheumatologist, were starting their first s.c. anti-TNF biologic and had a pre-anti-TNF DAS28 score of ≥5.1. Participants’ written consent was obtained according to the Declaration of Helsinki. The study was approved by a multicentre ethics committee (COREC 04/Q1403/37).

### Clinical and demographic data collection

Demographic details including age, gender and marital status were collected. Data collected on other clinical disease characteristics included NSAID usage, concurrent DMARD usage and disease duration prior to receiving the first biologic DMARD. Data were collected at baseline (prior to anti-TNF initiation) and at 3 and 6 months after initiation and DAS28 values were recorded at each time point. The DAS28 response over 6 months was categorized according to the EULAR response criteria [16].

### Measures

#### Adherence

**Fig. 1:**
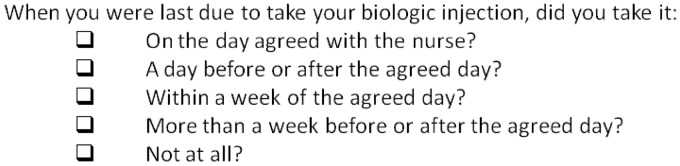
Adherence questionnaire

To assess adherence, a new behavioural self-report measure of adherence was designed by L.C. to assess the degree to which patients administered the biologic injection as advised by the prescribing health professional. Patients completed the measure at 3 and 6 months as shown in Fig. 1.

For the purposes of the current study, a classification of adherent was given if the injection was administered by the patient on the day agreed with the health care professional. Adherence behaviour was classified as ever non-adherent if it had been recorded as non-adherent at either or both of the 3 or 6 month data collection points.

#### Disease activity

The DAS28 was assessed at all time points using the DAS28 calculated according to standard practice [17].

### Statistical analysis

Multivariate linear regression was conducted to investigate the adherence status, demographic and clinical factors that affect change in DAS28 between baseline and 6 months. Potential confounders were included as independent variables in the model and included age, NSAID usage, DMARD usage, marital status, disease duration and baseline DAS28. In addition, the impact of adherence status and change to the individual components of the DAS28 after 6 months of treatment were investigated. Chi-squared test was used to investigate the variation in non-adherence rates between the different anti-TNF drugs assessed and the effect of non-adherence on EULAR response criteria. Continuous variables are presented as mean (s.d.) if normally distributed and median [interquartile range (IQR)] if non-normally distributed. These analyses were performed using Stata version 11.2 (StataCorp, College Station, TX, USA) [18].

## Results

### Patient characteristics

**Fig. 2:**
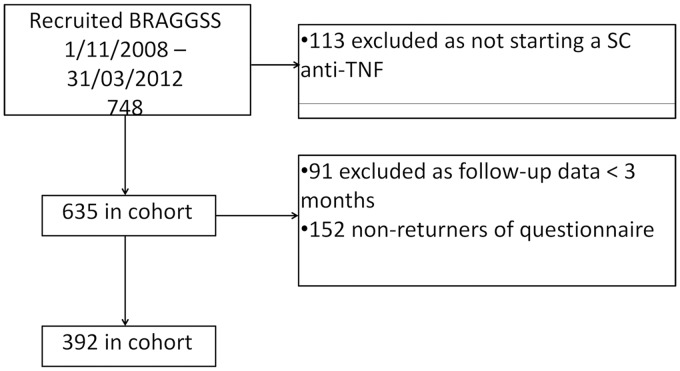
Flow chart showing recruitment of study participants

A total of 748 RA patients were registered from November 2008 to March 2012 within the prospective arm of the BRAGGSS cohort. Patients were excluded if they were not starting a s.c. anti-TNF biologic (*n* = 113) or had not yet reached 3 months of follow-up and thus had no follow-up DAS28 recorded (*n* = 91). A total of 152 (28%) patients did not return a patient questionnaire (Fig. 2). The final sample cohort totalled 392 RA patients, as shown in Table 1. Nearly 51% were co-prescribed NSAIDs when required or on a regular basis and 86% were prescribed concomitant DMARD therapy. Disease activity at baseline was high [median DAS28 5.94 (IQR 5.45–6.55)], with a mean DAS28 improvement of 2.73 (IQR 3.66–1.75) experienced after 6 months of s.c. anti-TNF therapy (Table 1).

**Table 1:** Demographic and clinical characteristics of the final sample cohort

Characteristic	Value
Age, median (IQR), years	58 (50.2–64.5)
Female, *n* (%)	292 (74.62)
Disease duration, median (IQR), years	7 (3.0–15.0)
Concurrent DMARD, *n* (%)	336 (85.7)
NSAID use, *n* (%)	197 (50.5)
Etanercept, *n* (%)	168 (42.9)
Adalimumab, *n* (%)	183 (47.1)
Certulizumab, *n* (%)	38 (9.7)
Golimumab, *n* (%)	1 (0.3)
Baseline DAS28, median (IQR)	5.94 (5.45–6.55)
3 month DAS28, median (IQR)	3.56 (2.49–4.78)
6 month DAS28, median (IQR)	3.21 (2.39–4.26)
6-month change in DAS28	−2.73 (−3.66 to −1.75)

IQR: interquartile range; DAS28: 28-joint DAS.

### Adherence

Table 2 presents self-reported adherence at 3 and 6 months and ever non-adherent frequency. Seventy-two per cent of those returning the questionnaire completed the adherence question. For those with complete data, adherence remained stable at 3 and 6 months (84.7% *vs* 84.5%, respectively). In total, 27% recorded that they were ever non-adherent during the 6-month study period. There was no difference in non-adherence rates between the different s.c. anti-TNF drugs assessed (*P* = 0.739, chi-squared test).

**Table 2:** Self-reported adherence at 3 and 6 months and ever non-adherent frequency

Degree of adherence	3 months, *n* (%)	6 months, *n* (%)	Ever non-adherent, *n* (%)
Day agreed upon	283 (84)	227 (85)	209 (73)
Day before or after	26 (8)	19 (7)	77 (27)
Within 1 week	16 (5)	6 (2)	
>1 week	6 (2)	4 (1)	
Not at all	4 (1)	12 (4)	
			

### Adherence and response to therapy

Table 3 presents the results of the multivariate linear regression indicating factors associated with changes in DAS28 score after 6 months of treatment with s.c. biologic therapy. Non-adherence was significantly associated with a lower DAS28 response following 6 months of s.c. anti-TNF therapy [β coefficient = 0.48 (95% CI 0.10, 0.86), *P* = 0.013]. Adherence was significantly associated with EULAR response (*P* = 0.015; Table 4), with a higher proportion of non-adherers defined as non-responders by the EULAR response criteria. Non-adherence was strongly associated with smaller changes in ESR after controlling for baseline ESR [β coefficient = 7.2 (95% CI 2.71, 11.67), *P* = 0.002, data not shown]. On evaluating whether answering the adherence question predicted response to treatment, there was no significant difference between question completers and non-completers [β coefficient − 0.01 (95% CI − 0.36, 0.34), *P* = 0.949].

**Table 3:** Multivariate linear regression results investigating factors associated with change in DAS28 score after 6 months of treatment with s.c. anti-TNF therapy

Demographic or clinical characteristic	β coefficient (95% CI)	*P*-value
Ever non-adherent	0.47 (0.10, 0.85)	**0.014**
Female	0.33 (−0.05, 0.72)	0.084
Age at baseline	0.02 (0.01, 0.04)	**0.002**
NSAID usage	−0.10 (−0.44, 0.19)	0.561
DMARD usage	−0.19 (−0.67, 0.29)	0.446
Married/living with partner	−0.20 (−0.59, 0.19)	0.307
Disease duration	−0.01 (−0.03, 0.01)	0.203
Baseline DAS28	−0.74 (−0.95, −0.53)	**<0.001**

Bold indicates statistical significance. DAS28: 28-joint DAS.

**Table 4:** Self-reported non-adherence to anti-TNF therapy by EULAR response

EULAR response	Non-adherent, *n* (%)	Adherent, *n* (%)	Total sample, *n* (%)
No response	12 (19)	12 (7)	24 (10)
Moderate response	27 (42)	71 (41)	98 (41)
Good response	25 (39)	92 (53)	117 (49)

*P* = 0.015. EULAR: European League Against Rheumatism.

## Discussion

In people with long-term conditions, a major challenge is optimizing patient adherence to therapy. In a group of patients with RA from the UK, our study showed that ∼27% of patients report being ever non-adherent during the first 6 months of starting a biologic. Importantly, the non-adherent group demonstrated a lower response to anti-TNF biologic therapy, even though the criterion used to classify non-adherence was strict. To our knowledge this is the first study to investigate self-reported adherence to s.c. anti-TNF biologics and to explore how this affects response to therapy.

We utilized a brief self-report measure of adherence that was quick and simple to administer. The acceptability of the question was good, with 72% of those returning the questionnaire completing the question. We report higher adherence to biologics compared with other published studies that utilize prescription claims data. There are a number of potential explanations for this finding. First, it is recognized that self-reported adherence tends to produce higher adherence estimates when compared with direct measures of behaviour, either because of recall difficulties or as a result of deliberate concealment of actual behaviour [19]. The wording of questions can have a significant impact on the response a patient gives. Questions that include statements such as ‘I was unable to do what was necessary to follow my doctor’s treatment plans’ will unsurprisingly produce higher rates of adherence [20]. To limit this type of social desirability effect we developed a new measure of self-reported adherence with deliberately neutral language. This measure utilized a timeline approach for assessing adherence in order to detect patient behaviour that did not correspond fully with the directions they had received. The intention was that partial non-adherers would feel comfortable answering that they took their drug a day before or after that agreed. While this may or may not absolutely reflect the patients’ actual behaviour in terms of timing of self-injection, it nonetheless was an indicator that the patient had not adhered fully. The association of non-adherence with a reduced response to therapy provides strong support for the predictive validity of this measure. The overall non-adherence rate of 27% is similar to previous research showing consistent self-reported non-adherence to all medication occurring in 24% of RA patients [21]. However, we were unable to include a biologic measure of adherence, e.g. drug levels, because the timing of the anti-TNF administration in relation to the blood sampling was not recorded. Second, previous studies of adherence have been performed in the USA, where there are fewer restrictions on the prescription of biologic drugs. Given that the eligibility criteria for prescription of biologic drugs for UK patients is non-response to two previous DMARDs plus high disease activity, higher rates of adherence might have been expected. It will be interesting to investigate the same adherence question in other countries with similar eligibility criteria to the UK to identify those contextual factors that have the greatest influence. Finally, prescription claims data may underestimate adherence because temporary stoppage of a biologic as instructed by a health care provider for intercurrent illness would be regarded as non-adherence.

The study has also demonstrated that the response to s.c. anti-TNF therapy is improved in patients who are younger and with high baseline DAS28 activity, similar to findings reported elsewhere [22, 23].

A major strength of this study is that we were able to investigate the association between adherence and response to therapy. The study has shown that patients who reported not administering their s.c. anti-TNF therapy on the day agreed have a poorer DAS28 response compared with those who do. This result strengthens the importance of close adherence to biologic therapies, as a DAS28 response ≥1.2 at 6 months is required to be able to continue therapy. Non-adherence is therefore likely to be a significant cause of non-response to therapy, leading to a waste of scarce health care resources, potential progression of disease and consequent increased health care costs. Physicians should therefore explore patients’ adherence, especially in those who are poor responders to therapy, and utilize existing guidelines in the UK for this purpose [3]. The results presented demonstrate that adherence is a strong predictor of response, therefore future studies investigating predictors of response to therapy should be conducted in an adherent cohort to reduce potential confounding or should include a measure of adherence as a covariate.

There are a number of limitations to the study that are worth noting. Adherence was only assessed at two time points, which may not be representative of the adherence history. For example, more patients may have been classified as non-adherent if assessed over additional time points. Furthermore, non-adherent patients may record themselves as adherent. However, in both scenarios the influence of non-adherence on response by 6 months would have been attenuated: the fact that an association with response was still observed meant that non-adherence was an important predictor of response.

The current study was not adequately powered to investigate the effect of different levels of adherence on clinical response, and partial non-adherence may still produce some clinical benefit [24]. The interaction between adherence and response is complex and may be bi-directional. The study did not differentiate between non-adherence due to forgetting and intentional non-adherence due to factors such as the perceived effectiveness of medications, which is known to affect intentional non-adherence [25]. Again, the sample size limited our ability to explore this. Selection bias could exist, in that patients who reply to patient questionnaires may also be those who are more likely to adhere to their biologic and thus respond. We investigated this possibility by comparing response in those patients who did not respond to the questionnaire and those who did. No significant difference in response between the two groups was observed, indicating that selection bias was not a major influence in our dataset.

In summary, this study has demonstrated that there is a significant proportion of RA patients who report not taking their prescribed s.c. anti-TNF at the time agreed with their clinician. Non-adherence is associated with poorer response, independent of demographic and treatment-related clinical factors. We describe a new measure of adherence with positive predictive validity that correlated with treatment response. Physicians should be aware that lower adherence affects response and should emphasize the importance of medication adherence to their patients.

**Rheumatology key messages**

- 27% of patients self-reported non-adherence at least once within 6 months of starting s.c. anti-TNF therapy
- Non-adherent patients with RA have a significantly reduced response to subcutaneous anti-TNF therapy.

## Supplementary Material

Supplementary Data
