# Supplementary material for: Impact of inadequate adherence on response to subcutaneously administered anti-tumour necrosis factor drugs: results from the Biologics in Rheumatoid Arthritis Genetics and Genomics Study Syndicate cohort
Source: Rheumatology (Oxford). 2014 Sep 10;54(3):494–9. doi: 10.1093/rheumatology/keu358 (PMC4334684; doi:10.1093/rheumatology/keu358)
Supplement: Supplementary Data [file supp_54_3_494__index.html]

Impact of inadequate adherence on response to subcutaneously administered anti-tumour necrosis factor drugs: results from the Biologics in Rheumatoid Arthritis Genetics and Genomics Study Syndicate cohort — Impact of inadequate adherence on response to subcutaneously administered anti-tumour necrosis factor drugs: results from the Biologics in Rheumatoid Arthritis Genetics and Genomics Study Syndicate cohort — Supplementary Data 

# Impact of inadequate adherence on response to subcutaneously administered anti-tumour necrosis factor drugs: results from the Biologics in Rheumatoid Arthritis Genetics and Genomics Study Syndicate cohort

## Supplementary Data

files

**Files in this Data Supplement:**

- Supplementary Data - docx file
