# Supplementary material for: Impact of inadequate adherence on response to subcutaneously administered anti-tumour necrosis factor drugs: results from the Biologics in Rheumatoid Arthritis Genetics and Genomics Study Syndicate cohort
Source: Rheumatology (Oxford). 2014 Sep 10;54(3):494–9. doi: 10.1093/rheumatology/keu358 (PMC4334684; doi:10.1093/rheumatology/keu358)
Supplement: Supplementary Data [file supp_keu358_rhe-14-0446-File004.docx]

# Members of the Biologics in Rheumatoid Arthritis Genetics and Genomics Study Syndicate (BRAGGSS)

1. Cambridge University Hospitals NHS Foundation Trust – Addenbrookes Hospital - Prof H. Gaston.
2. Mid Staffordshire General Hospitals NHS Trust – Cannock Chase Hospital - Dr D. Mulherin.
3. The Leeds Teaching Hospitals NHS Trust – Chapel Allerton Hospital – Prof P. Emery and Dr Maya Buch.
4. Derby Hospitals NHS Foundation Trust – Derbyshire Royal Infirmary – Dr S. O’Reilly.
5. Doncaster And Bassetlaw Hospitals NHS Foundation Trust – Doncaster Royal Infirmary – Dr R. Stevens.
6. Peterborough and Stamford Hospitals NHS Foundation Trust – Edith Cavell Hospital – Dr N. Williams.
7. The Newcastle upon Tyne Hospitals NHS Trust – Freeman Hospital – Prof J Isaacs.
8. University Hospital of North Staffordshire NHS Trust – Haywood Hospital (Stoke-on-Trent) – Dr A. Hassell.
9. Hereford Hospitals NHS Trust – Hereford County Hospital – Dr R Williams.
10. Norfolk & Norwich University Hospital NHS Trust – Norfolk & Norwich University Hospital – Dr C. Mukhtyar.
11. Pennine Acute Hospitals NHS Trust – North Manchester General Hospital – Dr L Das.
12. Portsmouth Hospitals NHS Trust – Queen Alexander Hospital – Dr J. Ledingham.
13. Gateshead Health NHS Trust – Queen Elizabeth Hospital – Dr C. Kelly.
14. Sheffield Teaching Hospitals NHS Trust – Royal Hallamshire Hospital – Dr M. Akil
15. University Hospital of Morcambe Bay NHS Trust – Royal Lancaster Infirmary – Dr M. Bukhari.
16. Sandwell and West Birmingham Hospitals NHS Trust – Sandwell General/City Hospital – Prof C. Buckley.
17. University Hospital Birmingham NHS Foundation Trust – Queen Elizabeth Hospital – Dr A Filer.
18. St Helens and Knowsley Hospitals NHS Trust – St Helens Hospital – Dr R. Abernathy.
19. South Tees Hospitals NHS Trust – The James Cook University Hospital, Middlesbrough – Dr M. Plant.
20. County Durham and Darlington Acute Hospitals NHS Trust – University Hospital of North Durham – Dr M. Bridges.
21. Whipps Cross University Hospital NHS Trust – Whipps Cross University Hospital – Dr A. Hakim.
22. The West Suffolk Hospital NHS Trust – West Suffolk Hospital – Dr D. O’Reilly.
23. Southampton University Hospital NHS Trust – Southampton General Hospital – Dr C. Edwards.
24. Basingstoke & North Hampshire NHS Foundation Trust – Basingstoke & North Hampshire Hospital – Dr P. Prouse.
25. Queen Mary's Sidcup NHS Trust – Queen Mary’s Sidcup (QMS) Hospital – Dr A. Bamji.
26. Pennine Acute Hospitals NHS Trust – Royal Oldham Hospital – Dr P. Klimiuk.
27. Pennine Acute Hospitals NHS Trust – Rochdale Infirmary – Dr A. Bowden.
28. University Hospitals of Morecambe Bay NHS Trust – Furness Hospital – Dr W. Mitchell.
29. Central Manchester University Hospital NHS Foundation Trust – Manchester Royal Infirmary – Prof I. Bruce.
30. The Dudley Group of Hospitals NHS Foundation Trust – Russells Hall Hospital – Prof G. Kitas.
31. Northumbria Healthcare NHS Foundation Trust – Wansbeck Hospital – Dr F. Birrell.
32. University Hospitals of Coventry and Warwickshire NHS Trust – University Hospital (was Walsgrave Hospital) – Dr M. Allen.
33. Wrightington, Wigan and Leigh Hospitals NHS Foundation Trust – Wrightington Hospital – Dr C. Chattopadhyay.
34. Nottingham University Hospitals NHS Trust – Nottingham Hospital – Dr J. McHale.
35. Salford Royal NHS Foundation Trust – Salford Royal Hospital – Dr H. Chinoy.
36. South Warwickshire General Hospital NHS Trust – Warwick Hospital – Dr C. Marguerie.
37. The Royal Bournemouth & Christchurch Hospitals NHS Foundation Trust – Christchurch Hospital – Dr N. Hopkinson.
38. Northern Lincolnshire and Goole Hospitals NHS Foundation Trust - Diana, Princess of Wales Hospital – Dr B. Szebenyi.
39. York Hospitals NHS Foundation Trust – York District Hospital – Dr M. Green
40. University Hospitals of Leicester NHS Trust – Leicester Royal Infirmary – Dr A. Samanta.
41. The Royal Wolverhampton Hospitals NHS Trust – New Cross Hospital – Dr W. Al-Allaf.
42. Greenpark Healthcare NHS Trust – Musgrave Park Hospital – Dr A. Taggart.
43. Chesterfield Royal Hospital NHS Foundation Trust – Chesterfield Royal Hospital – Dr K. Fairburn.
44. Central Manchester University Hospitals NHS Foundation Trust – Trafford General Hospital – Dr F. McKenna.
45. Harrogate and District NHS Foundation Trust – Harrogate District Hospital – Dr M. Green.
46. Royal National Hospital for Rheumatic Diseases NHS Foundation Trust – Bath Hospital – Dr J. Pauling.
47. Oxford Radcliffe Hospitals NHS Trust – John Radcliffe Hospital, Nuffield – Dr R. Luqmani.
48. Milton Keynes Hospital NHS Foundation Trust – Milton Keynes Hospital – Dr W. Smith.
49. Royal Liverpool and Broadgreen University Hospitals NHS Trust – Royal Liverpool Hospital – Dr D. Mewar.
50. Countess of Chester Hospital NHS Foundation Trust – Countess of Chester Hospital – Dr J. Nixon.
51. Royal Cornwall Hospitals NHS Trust – Royal Cornwall Hospital – Prof A. Woolf.
52. City Hospitals Sunderland NHS Foundation Trust – Royal Sunderland Hospital – Dr D. Coady.
53. Stockport NHS Foundation Trust – Stepping Hill Hospital – Dr I. Abbas.
54. Kettering General Hospital NHS Foundation Trust – Kettering General Hospital – Dr G. Kallarackal.
55. Burton Hospitals NHS Foundation Trust – Queens Hospital, Burton – Dr M. Nisar.
56. The Ipswich Hospital NHS Trust – Ipswich Hospital – Dr L. Shand.
57. Guy’s and St Thomas’ NHS Foundation Trust Guy’s Hospital – Dr A. Cope.
58. Pennine Acute Hospitals NHS Trust – Fairfield Hospital, Bury – Dr S. Naz.
